# In Vitro Digestion and Fermentation by Human Fecal Microbiota of Polysaccharides from Flaxseed

**DOI:** 10.3390/molecules25194354

**Published:** 2020-09-23

**Authors:** Xin Zhou, Zhao Zhang, Fenghong Huang, Chen Yang, Qingde Huang

**Affiliations:** Oil Crops and Lipids Process Technology National & Local Joint Engineering Laboratory, Hubei Key Laboratory of Lipid Chemistry and Nutrition, Key Laboratory of Oilseeds Processing, Oil Crops Research Institute of the Chinese Academy of Agricultural Sciences, Ministry of Agriculture and Rural affairs, No. 2 Xudong 2nd Road, Wuhan 430062, China; zhouxincaasgo@163.com (X.Z.); 13545367544@163.com (Z.Z.); huangfh@oilcrops.cn (F.H.); huangqd@oilcrops.cn (Q.H.)

**Keywords:** flaxseed polysaccharides, simulated digestion, gut microbiota, in vitro fermentation

## Abstract

The digestion of flaxseed polysaccharides (FSP) in simulated saliva, gastric and small intestine conditions was assessed, as well as in vitro fermentation of FSP by human gut microbiota. FSP was not degraded in the simulated digestive systems (there was no change in molecular weight or content of reducing sugars), indicating that ingested FSP would reach the large intestine intact. Changes in carbohydrate content, reducing sugars and culture pH suggested that FSP could be broken down and used by gut microbiota. FSP modulated the composition and structure of the gut microbiota by altering the *Firmicutes*/*Bacteroidetes* ratio and increasing the relative abundances of *Prevotella*, *Phascolarctobacterium*, *Clostridium* and *Megamonas*, which can degrade polysaccharides. Meanwhile, FSP fermentation increased the concentration of short-chain fatty acids, especially propionic and butyric acids. Our results indicate that FSP might be developed as a functional food that benefits gut health.

## 1. Introduction

The gut microbiota, consisting of numerous microbial species, is considered a vital organ that plays a pivotal role in host health [1]. Numerous studies have indicated that human gut microbiota can exert numerous benefits to the host health including nutrient absorption, glucose tolerance, and pathogen defense [2,3]. Growing evidence has demonstrated that plant polysaccharides cannot be digested by upper digestive tract, but can reach the distal intestine and be degraded by gut microbiota [4]. The fermentation of polysaccharides and their interaction with gut microbiota are closely related to human health. For example, polysaccharides extracted from soybean was found to promote the production of acetic acid and the relative richness of *Bifidobacterium*. In addition, polysaccharide fermentation can further improve the intestinal micro-ecosystem by inhibiting certain harmful bacteria in the host [5]. Recently, some scientists have proposed that using polysaccharides as a new therapeutic strategy to regulate gut microbiota for improving human health and preventing diseases [6,7].

Bioactive polysaccharide intake regulates the composition and metabolites of the gut microbiota [8]. Most polysaccharides modulate the diversity of gut microbiota by decreasing the ratio of *Firmicutes* to *Bacteroidetes*, which might be beneficial for regulating host lipid metabolism [9,10]. Polysaccharides could also protect against dysbiosis of the gut microbiota and inflammatory bowel disease by modulating the multiformity of the intestinal flora [11]. Moreover, the major metabolites arising from the microbial fermentation of dietary fiber, short-chain fatty acids (SCFAs), play a vital role in providing energy for epithelial cells [12] and maintaining the epithelial barrier. SCFAs can not only modulate immune response to prevent colorectal cancer, but also regulate the characteristics of metabolic syndrome [13,14].

Flaxseed (*Linum usitatissimum* L.) contains various of biological compounds such as n-3 fatty acids, good quality dietary fiber and bioactive lignans, which was considered as a good functional food ingredients [15,16]. The main soluble dietary fiber extracted from flaxseed is a heterogeneous polysaccharide, which consist of neutral arabinoxylan and acidic rhamnose [17]. Flaxseed polysaccharide (FSP) exhibited good antioxidant activities when scavenging radicals in vitro [18]. In addition, FSP intake can reduce the elevated blood glucose and bodyweight gain induced by high-fat diet in rats [19].

Our previous work demonstrated that FSP alleviated metabolic syndrome by modulating the gut microbiota and increasing the content of SCFAs in high-fat-diet mice [20]. However, there has been little study regarding the digestion and fermentation characteristics of FSP.

Therefore, in this study, in vitro digestion and fermentation models were conducted to investigate the digestion and fermentation traits of FSP. The interaction with gut microbiota and production of SCFAs owing to FSP fermentation were evaluated. This study provides a scientific basis for the bioactivity of FSP and its effects on promoting gut health.

## 2. Results and Discussion

### 2.1. Change of FSP on Digestion in Simulated Saliva

**Figure 1:**
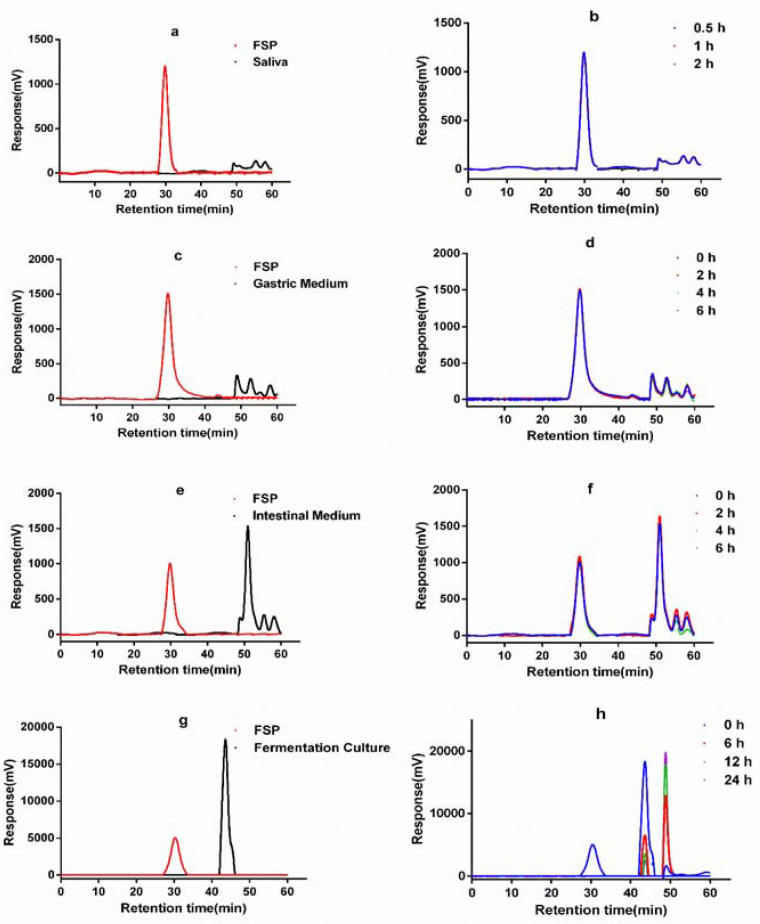
HPGPC chromatograms of FSP before and after saliva (**a**,**b**), simulated gastric (**c**,**d**), small intestinal (**e**,**f**) digestion and fermentation by human gut microbiota (**g**,**h**).

In the first stage of digestion, the primary function of the saliva is to convert food into small particles with the aid of alpha-amylase. Salivary α-amylase hydrolyses a-(1→4)-glycosidic bonds [21], facilitating the digestion of starch and other carbohydrates. Digestion of FSP by human saliva was studied in this work. As shown in Figure 1a,b, the molecular peak of FSP showed no significant change after saliva digestion, indicated by the retention time and response value. Furthermore, the C_R_ values of FSP solutions had no obvious difference before and after digestion (Table 1), showing that FSP was not hydrolyzed in saliva; this might be attributed to the structure of FSP. This may be due to the fact that the glycoside bonds in carbohydrates cannot be degraded by alpha-amylase [22]. It has been reported that FSP composed of at least 7 kinds of monosaccharides and can be divided into neutral polysaccharides and acidic polysaccharides parts. It has complex helical conformation and both α and β glycoside configurations [23], and thus was hard to be digested by α-amylase in saliva.

### 2.2. Change of FSP on Simulated Gastric Digestion

On treatment by simulated gastric juice, the retention time and response values (Figure 1c,d) of FSP were almost constant. There was no obvious change of FSP molecular weight during gastric digestion. The C_R_ content of FSP showed no change (Table 1). These data suggested that the gastric juice failed to degrade the FSP. The results of the current study were consistent with previously reported studies of polysaccharides from Fuzhuan brick tea [24]. However, it has been reported that gastric juice could lead to the degradation of polysaccharides from seeds of *Plantago asiatica* L. [25]. The digestibility and absorption characteristics of polysaccharides are influenced by their molecular weight, monosaccharide composition, glucoside bonding mode, and conformation. Our data indicated that the huge molecular weight and glucoside bonds of FSP hindered its degradation in saliva and gastric juice.

### 2.3. Change of FSP in Simulated Intestinal Digestion

The retention time indicating the molecular weight of FSP in the simulated small intestine juice was 30.2 min (Figure 1e). As shown in Figure 1f, both the molecular peaks of FSP and intestine medium did not change during 6 h incubation. The content of reducing sugars also remained unchanged (Table 1) during the simulated intestinal digestion. Thus, these results showed that the upper digestive tract had no effect on FSP. Here, the interaction between FSP and small intestinal microbiota was not considered. However, it is well known that the small intestine microbiota can also degrade and ferment diet-derived simple carbohydrates into organic acids, aldehydes, alcohols, and gases [26]. In vitro digestion is used to study the changes of FSP in the gastrointestinal juice, but it is difficult to study the effect of small intestinal microbiota on FSP. Thus, we focus on the degradation process of FSP in the fecal microbiota.

### 2.4. Change of FSP in In Vitro Fermentation by Human Gut Microbiota

**Figure 2:**
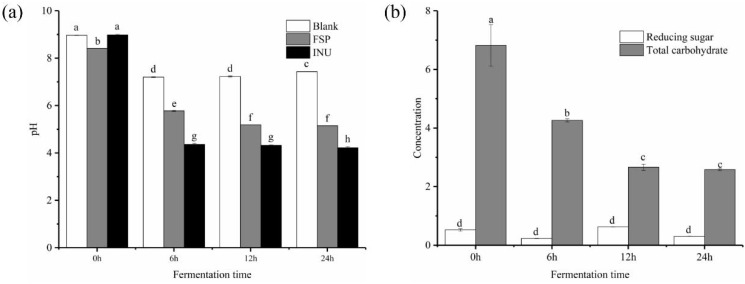
Shift of pH during in vitro fermentation (**a**) and change of contents of total carbohydrate and reducing sugar during fermentation (**b**). a–h means significantly different (*p* < 0.05) by a Tukey test in the same time between each two group.

Figure 1g showed the molecular peaks of FSP and fermentation medium. As displayed in Figure 1h, the peak of FSP disappeared after 6 h of fermentation, while peaks representing the FSP fragments appeared at 44.92 and 49.56 min, demonstrating that FSP was totally degraded into smaller fragments. As shown in Figure 2b, the initial concentrations of reducing sugar and total carbohydrate in FSP fermentation culture were 0.523 and 6.819 mg/mL, respectively. Thereafter, the concentration of reducing sugar increased to 0.625 mg/mL at 12 h and then began to decline, while the concentration of total carbohydrate decreased significantly from 0 to 12 h of fermentation

The degradation and use of polysaccharides by human fecal microbiota can lead to changes of monosaccharide composition [27]. We detected the residual monosaccharide composition of FSP at different fermentation time points. As shown in Table 2, after 24 h of fermentation, the percentages of xylose and arabinose were significantly increased. The amount of xylose increased rapidly, with a maximum content of 71.8%. The arabinose content reached 22.7%. The consumption rate of rhamnose was higher than that of other monosaccharides (decreased from 32.1% to 0% within 24 h). All the results demonstrated that FSP was degraded and used by gut microbiota.

The pH change of each group during anaerobic incubation reflects fermentation processes. As shown in Figure 2a, the pH value of all tested groups significantly (*p* < 0.05) declined from during fermentation process. In the FSP group, the pH decreased from 8.92 ± 0.01 to 5.78 ± 0.02 after 24 h fermentation and then remained stable. The pH of the INU group dropped sharply from 9.83 ± 0.01 to 4.36 ± 0.02 after 24 h fermentation, significantly lower than that of the FSP group. This may be due to the unequal amount of SCFAs produced by FSP and INU during the fermentation. Previous studies have shown that lowering the pH value in the gut lumen can inhibit the propagation of certain harmful microorganisms and improve the propagation of certain beneficial colonic microbiota [28,29].

**Figure 3:**
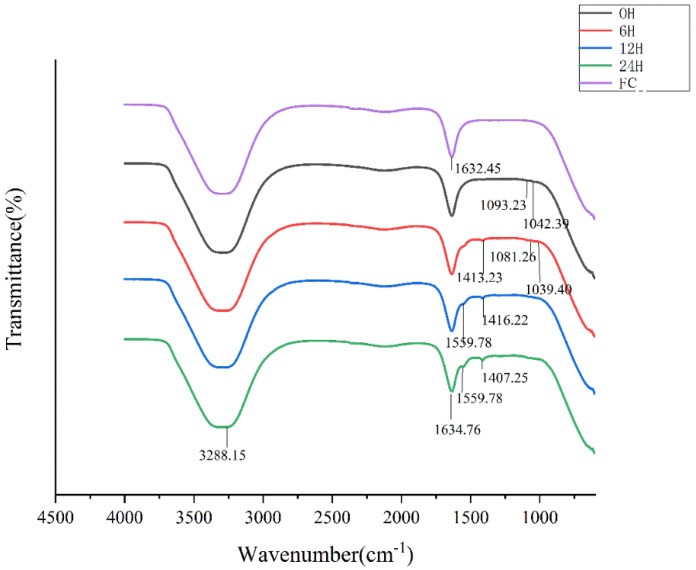
FT-IR spectra of FSP and its fermentation fractions (FC: Fermentation culture).

The FTIR spectrums of FSP fermentation fractions were illustrated in Figure 3. The broad absorption peak around 3400–3200 cm^−1^ was attributed to the stretching of O-H. This characteristic peak firstly moved toward the longer wavelength (3288 cm^−1^) after fermentation for 12 h and 24 h, compared with 6 h (3281 cm^−1^). the absorption peak at 1634 cm^−1^ might be stretching vibration of the deprotonated carboxylic group from amide I [30], indicating all fermentation fractions have glucosamine. After fermentation for 24 h, there existed some differences in absorption wavelength and intensity among different digestion phases. The absorption peaks centered at 1413 cm^−1^ was due to the presence of -CH2, which existed in the fermentation productions of 6–24 h, and not in fermentation productions of 0 h. The four absorption bands around 1100–1010 cm^−1^ were assigned to pyranose rings, representing characteristic peaks for carbohydrate [31], and the absorption peak disappears after 6 h of fermentation. Unique primary protein features found in peptide bonds include amide I and amide II (~60% N-H bending vibration, ~40% C-N stretching vibration; ca. 1550 cm^−1^). After fermentation for 12 h and 24 h, a new band appearing at 1559 cm^−1^ could be attributed to protein [32,33]. There is no such peak in the infrared results of pure FSP (data not shown); thus, this functional group for protein might indicated the production of protein metabolite by proliferated bacterium. Differences from 1413 cm^−1^ to 1039 cm^−1^ were attributed to the diversity of monosaccharide composition of fermentation fractions [34]. This result suggested the FSP was directly degraded by human fecal microbiota accompanied with the changes of polysaccharides structure and metabolite production of fecal microbiota.

### 2.5. Effects of FSP on SCFA Production

The effect of FSP on the intestinal environment was assessed by measuring the changes in SCFA concentrations during the fermentation process. Previous studies have demonstrated that SCFAs are considered as signaling molecules in miscellaneous cellular pathways. For instance, propionic acid can improve tissue insulin sensitivity and reduce fatty acid content in the liver [35]. Butyric acid is an energy source of intestinal epithelial cells, and exerts beneficial anti-inflammatory and anticarcinogenic effects [36].

The contents of individual and total SCFAs during in vitro fermentation of FSP are summarized in Table 3. Compared with the BLK group, the final concentration of total SCFAs increased 5.2- and 4.6-fold in the INU and FSP groups, respectively, illustrating that both FSP and inulin had a profound influence on production of SCFAs. Butyric, propionic, and acetic acids were the predominant products of FSP fermentation; the levels produced were close to those in the INU group and higher than those in the BLK group. Compared with the BLK group, propionic, butyric, isovaleric, and valeric acids were significantly higher in the FSP group at any fermentation time point (*p* < 0.05). In the INU group, the concentrations of propionic and *n*-butyric acid were a little higher than those in the FSP group, while the concentrations of isovaleric and valeric acid were much higher in the FSP group than those in the INU group. These results suggested that FSP and inulin probably promoted growth of different microorganisms producing different kinds of SCFA. The total SCFAs in the FSP group notably increased from 6 to 24 h, suggesting that FSP could slowly modulate the gut microenvironment.

### 2.6. Effects of FSP on Gut Microbiota

**Figure 4:**
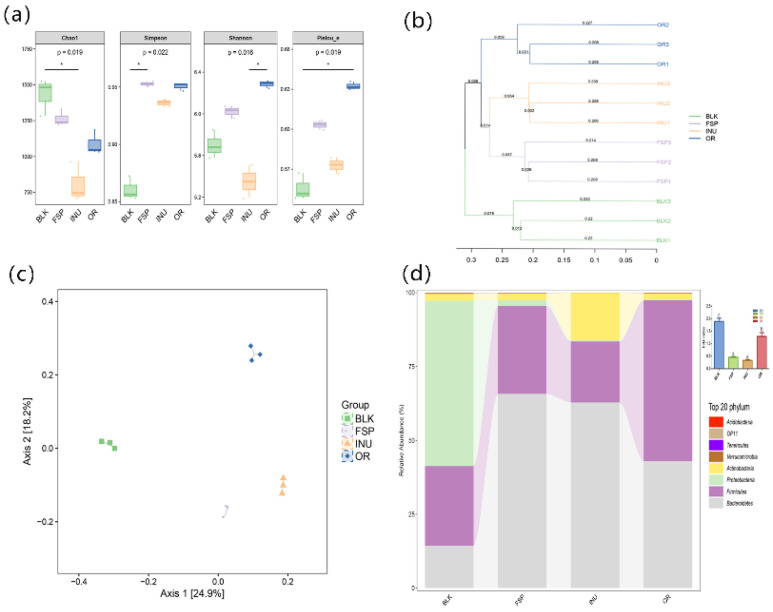
Compositions of gut microbiota. (**a**) Alpha diversity analysis of Simpson index and Shannon index, (**b**) Bray–Curtis clustering analysis, (**c**) principal component analysis of gut microbiota at the OTU level, (**d**) taxonomic composition distribution at the phylum level.

Figure 4a shows the alpha diversity of the gut microbiota community in each group. In this work, the Simpson, Shannon and Pielou indexes were significantly increased in the FSP group compared with the BLK group, indicating that degradation of FSP increased the microbial evenness. However, both FSP and INU treatment failed to elevate the Chao1 index, which might be because some bacterial species that could use the polysaccharide was enriched in the fecal culture, reduces the uniformity of microbial community. Hierarchical clustering showed that the FSP group formed a distinct cluster from the BLK and INU groups (Figure 4b). Principal coordinate analysis (PCoA) was performed to reveal the beta diversity of tested samples. As shown in Figure 4c, there was distinct separation among the four test groups. The first two axes interpreted 43.1% of the total variance (PC1: 24.9% and PC2: 18.2%), suggesting that the microbial community structure was different among the four groups.

FSP modulated the gut microbiota at the phylum level (Figure 4d). *Bacteroidetes* and *Firmicutes* were predominant bacterial phyla in the FSP and INU groups. The BLK group contained an average 52% abundance of the phylum *Proteobacteria,* which was found to be significantly decreased in both the FSP and INU samples. The portion of *Bacteroidetes* significantly increased in the FSP group compared with the BLK group while *Firmicutes* displayed the opposite change. The ratio of *Firmicutes/Bacteroidetes* (F/B) was significantly decreased by FSP fermentation. The F/B ratio is widely considered to be associated with energy harvest, and can characterize the risk of obesity [37]. The decreased F/B ratio caused by FSP fermentation suggested that FSP might have a beneficial effect on host weight loss. It has been demonstrated that FSP decreased the F/B ratio and inhibited obesity in high-fat-diet mice [20]. Additionally, at genus level, fermentation with FSP exhibited a higher *Phascolarctobacterium* but lower *Bifidobacterium* and *Faecalibacterium* in comparison with INU groups (Table 4). A significant growth in the genera *Megamonas* was observed in the FSP and INU groups.

**Figure 5:**
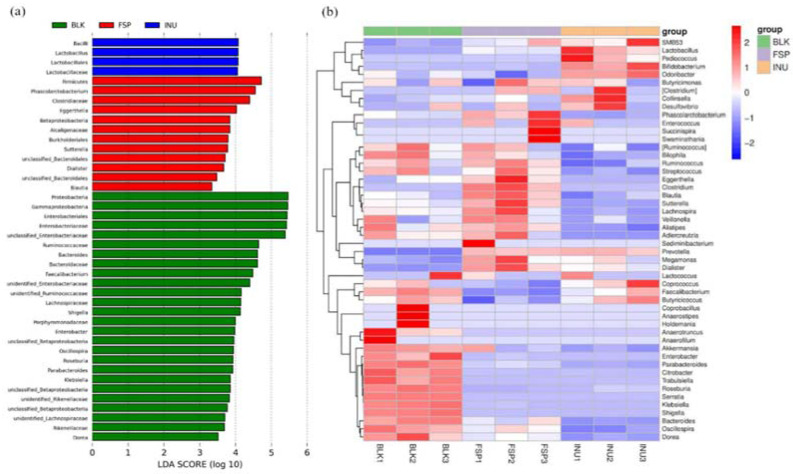
Comparisons of intra-group variance at OTUs level using LefSe. (**a**) LDA scores computed for features at OTUs level; (**b**) heatmap of the relative abundance of OTU (log 10 transformed).

Comparison of variance in relative abundance of gut microbiota was carried out by LEfSe analysis (used to analyze OTUs with relative abundance >0.1%). This demonstrated that a total of 42 OTUs were significantly different in the three groups (Figure 5a). Based on LDA values, 26 OTUs were abundant in the BLK group, 4 OTUs were abundant in the INU group, while 12 OTUs were abundant in the FSP group.

Key genera were notably influenced by FSP and INU treatments compared with the BLK group (Figure 5b). *Parabacteroides*, *Shigella*, *Bacteroides*, *Dorea*, *Citrobacter*, and *Roseburia* were enriched in the BLK group, but this enrichment was not observed in the INU and FSP groups. Notably, *Phascolarctobacterium*, *Clostridium*, *Sutterella*, *Ruminococcus* and *Blautia* were enriched in the FSP group. Among them, *Ruminococcus* and *Blautia* are SCFA-producing bacteria; they can degrade and use dietary fiber such as cellulose [38].

Composition of the top 20 taxon identified in the tested groups at genus level is shown in Table 4. The relative abundance of *Prevotella* increased from 3.12% to 60.39% (*p* < 0.001) and 61.38% (*p* < 0.001) in the FSP and INU samples, respectively, suggesting that *Prevotella* might be the principal gut microbiota that degrade and use polysaccharides. Increasing evidence has suggested that *Prevotella* are beneficial gut microbiota that can improve glucose metabolism [38]. The bloom of *Prevotella* by FSP suggested it could be used in maintain blood glucose stability in body as reported in mice [20] and rats [39]. *Dialister* was also stimulated by FSP and inulin both, it has been reported to use pectin as a substrate [40] and produce SCFAs [41].

Recently, Sutterella spp. was demonstrated to be involved in the adhesion of gut microbes to intestinal epithelial cells, and have the potential to regulate immune [42]. Moreover, *Phascolarctobacterium* was proved to be associated with propionate production through the succinate cycle [43]. This might explain the higher concentration of propionate observed in the FSP group. *Clostridium* was also detected at comparatively high relative abundance in the FSP group compared to BLK group. Based on previous reports, *Clostridium* can use various polysaccharides in versatile ways [44]. In addition, after fermentation, *Bifidobacterium* and *Lactobacillus* were significantly (*p* < 0.05) increased in the INU group compared with the BLK group. This result is consistent with previous reports that inulin could promote the proliferation of *Bifidobacterium* and *Lactobacillus* [28]. However, these two genera were not increased by FSP fermentation, indicating that FSP displayed a different pattern of regulation of the gut microbiota compared with inulin. Altogether, the fermentation of FSP modulated the microbial community structure and increased the microbial diversity, suggesting that FSP has potential prebiotic potency.

## 3. Materials and Methods

### 3.1. Materials

Golden flaxseed was purchased from Zhangye, Ganshu Province, China. Pancreatin, vitamin K and monosaccharide standards (including ribose (Rib), xylose (Xyl), mannose (Man), galacturonic acid (GalA), glucose (Glc) and galactose (Gal)) were purchased from Sigma Chemical Co. (St. Louis, MO, USA.) Bile salt was obtained from Solarbio Science & Technology Co.Ltd. (Beijing, China). SCFAs standards were purchased from Aladdin Chemical Reagent Co., Ltd. (Shanghai, China). All other chemicals were analytical grade.

### 3.2. Preparation of Polysaccharide

FSP was extracted from raw flaxseed using the method of Romain et al. [45] with modification. Firstly, the golden flaxseed was cleaned with distilled water. Then, flaxseeds were dissolved with distilled water (1:10, *w*/*w*) and stirred at 600 rpm at 60 °C for 2 h. The extracts were centrifuged at 4000 rpm for 10 min at 25 °C. The supernatants were concentrated with three volumes of 95% ethanol at 4 °C overnight to precipitate the polysaccharide. The polysaccharide was then collected by centrifuging at 5000 rpm for 15 min and finally dried in the freeze-dryer.

### 3.3. Simulated Saliva Digestion

The simulated saliva digestion of FSP was prepared following the reported method [25] with slight modification. First, FSP was dissolved in distilled water at 5 mg/mL concentration. Then, 4.0 mL of FSP solution (FSP group) was mixed with 4.0 mL of saliva and incubated in water bath oscillator (160 rpm, 37 °C). Equal volumes of distilled water (BLK group) and inulin (INU group) were used as blank control and positive control, respectively. During the digestion (at 0.5, 1, 1.5 and 2 h), 1.0 mL mixture was withdrawn, boiled in water bath for 5 min to deactivate salivary amylase. Each sample was replicated three times.

### 3.4. Simulated Gastric Digestion

The simulated gastric digestion was conducted as described the study by Fu et al. [46]. The gastric electrolyte solution (GES) was composed of 1.55 g NaCl, 0.55 g KCl, 0.075 g CaCl_2_·2H_2_O and 0.3 g NaHCO_3_ in 500 mL distilled water, and the solution was adjusted the pH to 2.0 by 0.1 M HCl solution. Then, gastric lipase (25.0 mg), gastric pepsin (23.6 mg) and 1 M CH_3_COONa (2 mL, pH 5.0) were added to 1 L of GES, affording the simulated gastric juice. After that, 50.0 mL of FSP solution (5.0 mg/mL) was prepared with equal volume gastric juice and incubated in water bath (37 °C, 160 rpm), while the control group was treated in a similar manner. After 0, 2, 4, and 6 h, samples (5 mL) were separately taken out and then deactivated enzymes for 5 min. Each experiment was replicated independently three times.

### 3.5. Simulated Small Intestinal Digestion

Simulated intestinal digestion of FSP was conducted as previous reports by Tedeschi et al. [47]. Briefly, the simulated small intestinal electrolyte solution (IES, 300 mL) involving 1.62 g NaCl, 0.195 g KCl and 0.099 g CaCl_2_•2H_2_O was prepared. Then, 50 g pancreatin solution (7%, *w*/*w*), 100.0 g bile salt (4%, *w*/*w*) and 6.5 mg trypsin were added into IES, and the pH was adjusted to 7.0. Subsequently, the digested gastric digestion (after 6 h) was mixed with the small intestinal juice at a ratio of 10:3. The resulting mixture was incubated in water bath oscillator (160 rpm) at 37 °C for 6 h. During the digestion of 0, 1, 2, 4 and 6 h, samples (5 mL) were separately taken out and then deactivated enzymes for 5 min.

### 3.6. In Vitro Fermentation of FSP

#### 3.6.1. Preparation of Fermentation Medium

The basal medium was conducted in accordance with the report by Ding et al. [48]. Briefly, 500 mL of basal medium consisted of 1.0 g peptone, 1.0 g yeast extract, 0.025 g hemin, 0.25 g _L_-cysteine, 0.25 g bile salts, 0.05 g NaCl, 0.02 g K_2_HPO_4_, 0.02 g KH_2_PO_4_, 0.005 g MgSO_4_, 0.005 g CaCl_2_, 1 g NaHCO_3_, 0.5 mg resazurin, 1.0 mL Tween-80, and 5 μL vitamin K. After pH was adjusted to 7.0, the fermentation medium was autoclaved at 12 °C for 20 min.

#### 3.6.2. Preparation of Fecal Slurry and Fermentation

Fecal samples were pooled from three healthy volunteers (one male and two females, age 20–30) who remained on regular diet and had not used antibiotics in at least 3 months. Males should weigh no less than 55 kg and women should weigh no less than 45 kg. Volunteers should be in good health and have no history of heart, liver, kidney, digestive tract, nervous system, mental disorders, and metabolic disorders. Firstly, fresh fecal samples were collected in a triangle beaker, mixed and dispersed with 10% sterilized PBS to obtain a 20% (*w*/*v*) feces suspension. Feces suspension was filed with three layers of gauze to obtain fecal slurry. Then, 7.0 mL of the fecal slurry was added into 28.0 mL of basal medium containing 350.0 mg FSP or inulin (INU), or not (BLK), respectively. The entire transport and mix process are completed in a clean bench to ensure no contamination. The fermentation process was carried out in an anaerobic tank (Mitsubishi Chemical, Japan) to ensure an aerobic environment. All groups were incubated at 37 °C. Samples were taken out after fermentation at 6, 12 and 24 h for future analysis. All experimental protocols were approved by the Ethical Committee of the faculty of Hubei Biosafety (China, 2019.08.10). All enrolled subjects were able to comply with the study procedures and gave their written informed consent prior to initiation of the trial. Throughout the study, the principles of Declaration of Helsinki and further amendments were fully respected.

### 3.7. Determinations of pH, Carbohydrate Content and Reducing Sugar

The pH value was measured by a pH meter (Mettler-Toledo Instruments Co., Ltd., Shanghai, China). The total carbohydrate content was measured by the phenol–sulfuric acid method [49] using glucose as standard. The contents of reducing sugar (C_R_) were evaluated by dinitro salicylic acid (DNS) method [50].

### 3.8. Determination of SCFA Content

The content of SCFAs was analyzed according to the reported method [51]. In brief, the fermentation products were centrifuged at 4500 rpm for 10 min and filtered through a 0.22 μm membrane filter to obtain supernatant before analysis. Then, the supernatant of 200 ul was mixed with 0.5 uL 2-ethylbutyric acid as internal standard to determine the SCFA_S_. SCFAs determination was implemented on GC (7890N, Agilent, Santa Clara, CA, USA),) equipped with a DB-FFAP column (30 m × 0.53 mm× 0.50 μm, Agilent) and flame ionization detector. The carrier gas was N_2_ with split ratio of 1:10. The initial temperature of column was 80 °C, maintained for 0.5 min and increased to 180 °C at a rate of 8 °C/min. The detector temperature was finally kept at 200 °C for 5 min. The SCFAs concentration was analyzed based on standard calibration curves.

### 3.9. DNA Extraction and Analysis

After fermentation for 24 h, total genomic DNA from fresh slurry (OR) group, BLK group, FSP group and INU group were immediately extracted by using the QIAamp DNA Stool Mini Kit [52]. The V4 region of 16S rRNA of each sample was chosen for amplification and sequenced by Personalbio Bio. Technology (Shanghai, China) Co., Ltd. Bioinformatics analysis mainly included principal component analysis (PCA), cluster analysis and linear discriminant analysis effect size (LEfSe).

### 3.10. Molecular Weight Determination

The molecular weight of FSP was determined by HPGPC according to the reported method [53]. The chromatographic separation was carried out by a BRT105-104-102 column and Gel column (8 × 300 mm, borui Saccharide, Biotech, Yangzhou, China. Co. Ltd.). The mobile phase was 0.05 M NaCl solution (0.6 mL/min). The column temperature was maintained at 35 ± 0.2 °C. The sample were centrifuged at 12,000 rpm for 10 min, and fermentation products filtered through a 0.22 μm membrane filter before injection. The injection volume was 20 μL. The standard curve was drawn with Dextran standards (1152 Da, 11,600 Da, 23,800 Da, 48,600 Da, 80,900 Da, 148,000 Da, 273,000 Da, 409,800 Da).

### 3.11. Determination of Free Monosaccharide and Fourier Transform Infrared (FT-IR) Spectra

FSP was added into 3 mol/L trifluoroacetate and hydrolyzed at 110 °C for 4 h. The hydrolysate was then derived with 200 uL sodium carbonate, anhydrous methanol (3–5 mL) according to a previously described method [54]. Analysis was performed on shimadzu GC-MS-QP 2010 (Shimadzu Technologies, Kyoto, Japan). Samples were isolated with RXI-5 SIL MS column (30 m × 0.25 mm × 0.25 μm, Shimadzu). The initial column temperature was 250 °C, held for 15 min. The pressure was 73.0 kPa, the detector temperature was 290 °C. The carrier gas was helium and the flow rate was 1 mL/min. Standard sugars were determined in the same way. The monosaccharide composition in the sample was obtained by comparing it to the HPLC result of standard sugar.

The fermentation time of 0, 6, 12, and 24 h, 1.0 mL of fermentation supernatant was taken out and placed in a diamond ATR module for infrared (FT-IR) spectrometry. The FT-IR spectra were recorded on a Nicolet 6700 FT-IR spectrometer (Thermo Fisher Scientific, Inc., Waltham, MA, USA) in the frequency range of 600–4000 cm^−1^ at 4 cm^−1^ resolution using 32 scans for three or more times.

### 3.12. Statistical Analysis

All the results were expressed as mean ± standard deviations and all estimations were run in triplicate. The results were analyzed using SPSS 25 software by a Tukey′s test. Values were considered to be statistically significant at *p* < 0.05.

## 4. Conclusions

FSP cannot be degraded by simulated saliva or gastric or small intestine conditions, so it reaches the large intestine intact. FSP was gradually hydrolyzed and used by human fecal microbiota during fermentation. FSP fermentation exhibited a modulatory effect on the gut microbial community structure by reducing the ratio of *Firmicutes* to *Bacteroidetes*. FSP stimulated the growth of beneficial and SCFA-producing bacteria such as *Prevotella* and *Phascolarctobacterium*. Moreover, the content of SCFAs, especially acetic and propionic acids, was significantly increased during FSP fermentation. Therefore, all the results suggest that FSP might contribute to gut health and could be a candidate for use as a prebiotic in functional foods.

## Figures and Tables

**Table 1:** Reducing sugar of FSP during digestion of saliva, gastric and small intestine.

Process	Time (h)	Content of Reducing Sugars (mg/mL)
Saliva digestion	0	0.236 ± 0.009 ^a^
	0.5	0.227 ± 0.005 ^a^
	1	0.234 ± 0.006 ^a^
	1.5	0.235 ± 0.008 ^a^
	2	0.233 ± 0.006 ^a^
Gastric juice digestion	0	0.116 ± 0.011 ^a^
	1	0.117 ± 0.012 ^a^
	2	0.115 ± 0.012 ^a^
	4	0.132 ± 0.014 ^a^
	6	0.118 ± 0.003 ^a^
Intestinal juice digestion	0	0.532 ± 0.301 ^a^
	1	0.545 ± 0.129 ^a^
	2	0.565 ± 0.203 ^a^
	4	0.54 ± 0.178 ^a^
	6	0.549 ± 0.219 ^a^

^a^ means no significantly different (*p* < 0.05) by a Tukey test in the same column with different letter.

**Table 2:** Compositions and contents of residual monosaccharides at different time points of fermentation.

Fermentation Time (h)	Monosaccharide (%)				
	Rha	Fuc	Ara	Xyl	Gal
0	32.1 ± 0.018 ^a^	5.6 ± 0.004 ^a^	12.4 ± 0.007 ^a^	39.4 ± 0.015 ^a^	10.4 ± 0.001 ^a^
6	20.0 ± 0.009 ^b^	3.4 ± 0.002 ^b^	15.9 ± 0.005 ^b^	53.0 ± 0.029 ^b^	7.6 ± 0.003 ^b^
12	3.0 ± 0.002 ^c^	0.6 ± 0.001 ^c^	21.5 ± 0.010 ^c^	68.1 ± 0.031 ^c^	6.8 ± 0.005 ^c^
24	0 ± 0.002 ^d^	0 ± 0.031 ^d^	22.7 ± 0.009 ^d^	71.8 ± 0.042 ^d^	5.6 ± 0.004 ^d^

a–d means the data showed significantly differences (*p* < 0.05) by a Tukey test within a column.

**Table 3:** The concentrations of SCFAs in fermentation solutions at different time points of fermentation.

Group	Time (h)	SCFAs (mmol/L)						
		Acetic Acid	Propionic Acid	i-Butyric Acid	n-Butyric Acid	i-Valeric Acid	n-Valeric Acid	Total
BLK	0	0.275 ± 0.012 ^b^	1.578 ± 0.015 ^a^	0.00 ± 0.00 ^a^	1.503 ± 0.050 ^b^	0.210 ± 0.011 ^a^	0.00 ± 0.00 ^a^	3.568 ± 0.062 ^a^
	6	0.904 ± 0.050 ^d^	2.054 ± 0.105 ^a^	0.164 ± 0.009 ^b^	1.689 ± 0.075 ^b^	0.254 ± 0.020 ^a^	0.297 ± 0.018 ^b^	5.362 ± 0.067 ^b^
	12	0.704 ± 0.055 ^c^	3.412 ± 0.086 ^b^	0.338 ± 0.062 ^c^	1.116 ± 0.075 ^a b^	0.592 ± 0.054 ^c^	0.886 ± 0.091 ^c^	7.051 ± 0.176 ^c^
	24	0.00 ± 0.00 ^a^	3.389 ± 0.093 ^b^	0.433 ± 0.030 ^d^	0.587 ± 0.041 ^a^	0.936 ± 0.112 ^d^	0.865 ± 0.111 ^c^	6.211 ± 0.103 ^bc^
INU	0	0.275 ± 0.012 ^b^	1.578 ± 0.015 ^a^	0.00 ± 0.00 ^a^	1.503 ± 0.050 ^b^	0.210 ± 0.011 ^a^	0.00 ± 0.00 ^a^	3.568 ± 0.062 ^a^
	6	2.027 ± 0.073 ^e^	10.093 ± 0.200 ^d^	0.00 ± 0.00 ^a^	7.051 ± 0.236 ^d^	0.208 ± 0.004 ^a^	0.323 ± 0.013 ^b^	19.702 ± 0.470 ^e^
	12	2.008 ± 0.040 ^e^	15.408 ± 0.510 ^f^	0.00 ± 0.00 ^a^	9.213 ± 0.352 ^e^	0.426 ± 0.011 ^b^	0.311 ± 0.018 ^b^	27.366 ± 0.895 ^f^
	24	4.150 ± 0.049 ^g^	15.029 ± 0.200 ^f^	0.00 ± 0.00 ^a^	12.271 ± 0.742 ^g^	0.631 ± 0.040 ^c^	0.386 ± 0.022 ^b^	32.467 ± 0.901 ^h^
FSP	0	0.275 ± 0.012 ^b^	1.578 ± 0.015 ^a^	0.00 ± 0.00 ^a^	1.503 ± 0.050 ^b^	0.210 ± 0.011 ^a^	0.00 ± 0.00 ^a^	3.568 ± 0.062 ^a^
	6	0.00 ± 0.00 ^a^	7.495 ± 0.526 ^c^	0.00 ± 0.00 ^a^	5.296 ± 0.318 ^c^	0.574 ± 0.061 ^c^	0.901 ± 0.074 ^c^	14.266 ± 0.881 ^d^
	12	2.569 ± 0.105 ^f^	14.053 ± 0.254 ^e^	0.00 ± 0.00 ^a^	10.484 ± 0.454 ^f^	1.508 ± 0.011 ^e^	2.111 ± 0.139 ^d^	30.723 ± 0.081 ^g^
	24	2.079 ± 0.088 ^e^	13.918 ± 0.475 ^e^	0.00 ± 0.00 ^a^	9.121 ± 0.236 ^e^	1.383 ± 0.033 ^e^	2.023 ± 0.177 ^d^	28.52 4 ± 0.081 ^f^

a–g: Mean values in the same column with different letters are significantly different (*p* < 0.05) by a Tukey test. SCFAs, short-chain fatty acids; BLK, blank group; INU, inulin; FSP, polysaccharides from Flaxseed.

**Table 4:** Composition of the top 20 taxons identified in the tested groups at genus level.

Taxon			Mean (%)			SEM (%)		
Phylum	Family	Genus	BLK	FSP	INU	BLK	FSP	INU
*Bacteroidetes*	*Prevotellaceae*	*Prevotella*	3.12 ^b^	60.39 ^a^	61.38 ^b^	0.39	1.44	3.89
	*Porphyromonadaceae*	*Parabacteroides*	1.68 ^a^	0.46 ^b^	0.14 ^c^	0.09	0.05	0.03
	*Bacteroidaceae*	*Bactericides*	9.20 ^a^	4.46 ^b^	1.10 ^c^	0.67	1.27	0.23
*Actinobacteria*	*Bifidobacteriaceae*	*Bifidobacterium*	2.24 ^b^	2.12 ^b^	15.83 ^a^	0.37	0.47	2.77
*Proteobacteria*	*Alcaligenaceae*	*Sutterella*	0.93 ^b^	1.69 ^a^	0.41 ^b^	0.11	0.41	0.04
*Firmicutes*	*Veillonellaceae*	*Phascolarctobacterium*	3.90 ^b^	8.76 ^a^	1.61 ^b^	0.67	2.29	0.36
	*Clostridiaceae*	*Clostridium*	0.16 ^b^	4.86 ^a^	0.15 ^b^	0.01	1.23	0.03
	*Veillonellaceae*	*Megamonas*	2.13 ^b^	4.73 ^a^	3.42 ^b^	0.28	1.10	0.53
	*Ruminococcaceae*	*Faecalibacterium*	7.83 ^a^	1.76 ^c^	5.63 ^b^	0.65	0.31	1.25
	*Ruminococcaceae*	*Oscillospira*	2.04 ^a^	1.35 ^b^	0.44 ^c^	0.22	0.19	0.13
	*Lachnospiraceae*	*Ui_Lachnospiraceae*	1.59 ^a^	1.26 ^a^	0.59 ^b^	0.16	0.16	0.12
	*Ruminococcaceae*	*Ui_Ruminococcaceae*	3.91 ^a^	1.28 ^c^	2.89 ^b^	0.17	0.08	0.57
	*Veillonellaceae*	*Dialister*	0.15 ^c^	0.97 ^a^	0.55 ^b^	0.02	0.23	0.07
	*Lachnospiraceae*	*Blautia*	0.48 ^b^	0.77 ^a^	0.35 ^b^	0.06	0.10	0.08
	*Lactobacillaceae*	*Lactobacillus*	0.01 ^b^	0.78 ^b^	2.27 ^a^	0.00	0.10	0.85
	*Ui_Clostridiales*	*Ui_Clostridiales*	0.42 ^a^	0.45 ^a^	0.17 ^b^	0.06	0.08	0.07
	*Ruminococcaceae*	*Uc_Ruminococcaceae*	0.44 ^a^	0.35 ^a^	0.40 ^a^	0.01	0.05	0.08
	*Lachnospiraceae*	*Uc_Lachnospiraceae*	0.25 ^a^	0.41 ^a^	0.37 ^a^	0.03	0.10	0.06
	*Ruminococcaceae*	*Ruminococcus*	0.27 ^a^	0.31 ^a^	0.16 ^b^	0.04	0.03	0.03
	*Uc_Clostridiales*	*Uc_Clostridiales*	0.31 ^a^	0.32 ^a^	0.16 ^b^	0.03	0.07	0.01

a–c means significantly different (*p* < 0.05) by a Tukey test in the same column with different letter.
